# Supplementary material for: Correlation of single-round strokes & performance indicators: Paris Olympic elite female golfers
Source: PLoS One. 2026 Jun 5;21(6):e0338350. doi: 10.1371/journal.pone.0338350 (PMC13240931; doi:10.1371/journal.pone.0338350)
Supplement: Attachment — Submitted filename: Response to reviewers.docx [file pone.0338350.s003.docx]

**Response Letter**

Manuscript ID: PONE-D-25-49380

Manuscript Title: *Correlation of Single-Round Strokes & Performance Indicators: Paris Olympic Elite Female Golfers*

Dear Academic Editor: Emiliano Cè, Ph.D. and Reviewer: Wissem Dhahbi,

Thank you for your letter and the reviewer’s constructive feedback on our manuscript. We sincerely appreciate the time and effort dedicated to evaluating our work, and we are grateful for the opportunity to revise and improve the manuscript. All comments have been addressed point-to-point below, with responses highlighted in red font. Moreover, we have indicated the specific locations of the corresponding changes in the revised manuscript.

We believe these revisions address all the points raised by the editor and reviewer, significantly improving the quality and clarity of our manuscript. Thank you again for your valuable input. We look forward to your favorable reconsideration of our submission.

Best regards,

The Corresponding Author

**Responses to Journal Requirements**

1.Style Requirements

***Re:*** We have carefully reviewed and adjusted the manuscript to comply with PLOS ONE’s style guidelines, including file naming conventions, using the templates provided at the specified URLs. All revised files are renamed according to journal guidelines.

2.The statements of not constitute dual publication

***Re:*** We appreciate the attention to the preliminary presentation of this work. Below is a detailed clarification regarding the prior conference publication and justification for non-duplicate submission:

The preliminary findings of this study were published in the 2025 6th Asia Sport Science Conference (Title: Analysis of the Competition Results of Elite Female Golfers at the Paris Olympics Using Multiple Linear Regression). This conference proceeding underwent peer review during the submission process (consistent with the conference’s review policy for academic validity), but it is categorized as a conference publication which not a formally peer-reviewed journal article. It was not indexed in core academic databases and did not undergo the rigorous editorial and review process required for journal publication.

The current manuscript represents a substantial extension and deepening of the preliminary conference work, with critical additions in methodology, data analysis, and research value that distinguish it as an independent study: The incorporation of principal component analysis (PCA) to better handle multicollinearity among performance indicators and extract latent factors, which was not included in the conference version.Expanded statistical analyses, including correlation analysis and more comprehensive model diagnostics. A more in-depth interpretation of results, with updated references and broader implications for training and competition strategy. A refocused introduction and discussion contextualizing the study within the scarce literature on Olympic female golfers, unlike the conference paper which had a narrower scope. Therefore, this submission does not constitute dual publication but rather represents a significant scholarly advancement suitable for publication in PLOS ONE.

We have added a statement that does not constitute a double publication in the "COVER LETTER" and re-uploaded the file (Lines 17-23).

3.Data Availability

***Re:*** In accordance with PLOS ONE's data availability policy, we have now uploaded the all raw data underlying our study as a Supporting Information file (labeled 'Supplementary Material 1') with the resubmitted manuscript. This ensures that all data are fully available without restriction to other researchers. Our data, which were sourced from the public domain of the official Olympics website, do not involve any ethical or privacy concerns that would preclude public deposition.

4.Participant privacy

***Re:*** We appreciate your critical reminder regarding the protection of participant privacy. In order to fully comply with PLOS ONE's data sharing policy and ethics requirements, we have modified the relevant content in the manuscript to delete all content in Table 1 and modify the content of the manuscript to:This study analyzed performance data from a total of 12 elite female golfers who finished within the top 10 ranks (including ties) in the Women's Individual Stroke Play at the 2024 Paris Olympics, resulting in 48 rounds (4 rounds per player). To protect participant privacy in accordance with journal policy, all personally identifiable information has been removed. The cohort comprised athletes from diverse geographical regions and physical profiles, representative of the international field at the Olympic Games (Line 123-129).

5.Suggestions to cite specific published works

***Re:*** We appreciate your thoughtful reminder. We will carefully review and evaluate the previously published works recommended in the reviewer comments to determine their relevance to the core content and research focus of this manuscript, and decide whether to include them as citations accordingly.

**Responses to Reviewer #1**

**General Comments**

***Re:*** We appreciate the reviewer's recognition of the timeliness and relevance of our topic and the clear articulation of our research question. We have taken the major weaknesses identified to heart and have implemented substantial revisions to the statistical methodology and clarity of definitions.

**Major Weaknesses**

①Sample Size and Statistical Power

***Re:*** We acknowledge your critical point regarding the sample size (N=48: 12 players × 4 rounds) and its potential impact on statistical stability. We emphasize that the sample size is inherently constrained by the unique nature of the research context: the 2024 Paris Olympic Women’s Golf Tournament featured only 60 athletes worldwide, and our study focused on the top 10 (including ties, 12 players)—the core cohort of elite performers whose performance patterns are most relevant to the research question. This sample is representative of the “Olympic elite” population (a scarce and highly selective group), and similar studies on Olympic-specific sports performance (e.g., elite gymnastics, or fencing) have adopted comparable sample sizes due to population limitations. [ Keskin, Ö. (2024). Comparison of Trampoline Gymnastics Athletes' Performance and Normative Data of Tokyo Olympic Games. International Journal of Sport Exercise and Training Sciences-IJSETS, 10(3), 184-194.Di Martino, G., Centorbi, M., Buonsenso, A., Fiorilli, G., Della Valle, C., Iuliano, E. & di Cagno, A. (2024). Assessing the impact of fencing on postural parameters: observational study findings on elite athletes. Sports, 12(5), 130. ]

Traditional power analysis frameworks are primarily designed for hypothesis testing rather than predictive modeling. For regression-based prediction, the critical considerations are model stability and generalizability rather than conventional power calculations. To address these concerns, we implemented several methodological safeguards: LASSO regression with L1 regularization: This approach inherently addresses overfitting by shrinking coefficients toward zero, effectively reducing model complexity and variance—a strategy specifically recommended for high-dimensional settings with limited samples. Leave-one-out cross-validation (LOOCV): LOOCV provides nearly unbiased prediction error estimates and is particularly advantageous for small samples compared to k-fold cross-validation. Bootstrap validation: This resampling procedure assessed variable selection stability, with seven predictors achieving≥90% selection frequency, indicating robust rather than spurious associations. Cross-validated MSE reporting: We report CV-MSE (0.528) alongside adjusted R² (0.928), providing a more conservative estimate of model generalizability. We have added explicit acknowledgment in the Limitations section that external validation in independent cohorts is warranted to confirm these findings (Line 547-552).

②Principal Component Analysis (PCA)

***Re:*** We fully agree with your critique and acknowledge two critical methodological errors in the original PCA implementation: (1) inclusion of the dependent variable (Single-Round Strokes) and (2) insufficient observations per variable. We have revised the PCA procedure comprehensively:

PCA was re-conducted using only the 13 predictor variables (X1-X13), consistent with standard dimensionality reduction practices where PCA should precede outcome modeling rather than incorporate the outcome itself.

Clearly stated this constraint in the Methods section Interpreted the extracted components with appropriate caution as exploratory rather than confirmatory. Supplemented PCA with LASSO regression, which is methodologically suited for situations where p approaches n. Components were retained based on eigenvalues≥1.0 and cumulative variance contribution ≥70%, yielding five principal components explaining 77.94% of total variance.

③Stepwise Multiple Linear Regression and Overfitting

***Re:*** We have replaced the stepwise multiple linear regression with a more statistically sound and theoretically driven approach. We have completely replaced stepwise regression with LASSO (Least Absolute Shrinkage and Selection Operator) regression, which offers several advantages for our data structure:

L1 regularization: Unlike stepwise regression, which performs discrete variable selection without penalization, LASSO simultaneously performs variable selection and coefficient shrinkage through the L1 penalty term. This approach reduces overfitting by constraining the total magnitude of coefficients, effectively trading increased bias for substantially reduced variance.

Leave-one-out cross-validation (LOOCV): The regularization parameter (λ) was optimized via LOOCV rather than relying on in-sample fit statistics. This provides a more honest estimate of prediction error, as each observation is iteratively held out for validation. The optimal λ = 0.094 was determined at minimum cross-validated MSE.

Bootstrap stability assessment: We performed 1,000 bootstrap iterations to evaluate variable selection stability. Seven of nine selected variables demonstrated high stability (selection frequency ≥90%): GIR (100%), Putt-Total (99%), SG: Approach the Green (98%), SG: Around the Green (97%), SG: Putting (94%), SG: Off the Tee (92%), and Scrambling (88%). This stability analysis confirms that the identified predictors reflect robust associations rather than overfitting artifacts.

Conservative performance reporting: We now report both adjusted R² (0.928) and cross-validated MSE (0.528). While adjusted R² reflects training set performance and may overestimate predictive accuracy, CV-MSE provides a more conservative and generalizable estimate. We explicitly acknowledge that external validation in independent samples is necessary to confirm model performance.

④Data Availability Statement

***Re:*** Uploading all anonymized raw data as Supplementary Materials 1, which complies with PLOS ONE’s data availability policy.

⑤Clarity of "Strokes Gained" (SG) Definitions

***Re:*** To avoid ambiguity and readability in SG, we have followed the official definition of the Paris Olympics.

SG: Off the Tee (*X3*): The number of strokes a player takes from a specific distance off the tee on par 4 and par 5 is measured against a statistical baseline to determine the player's strokes gained or lost off the tee on a hole. The sum of the values for all holes played in a round minus the field average strokes gained/lost for the round is the player's Strokes gained/lost for that round. The sum of strokes gained for each round are divided by total rounds played (Line 184-189).

SG: Approach the Green (*X6*): The number of Approach the Green strokes a player takes from specific locations and distances are measured against a statistical baseline to determine the player's strokes gained or lost on a hole. The sum of the values for all holes played in a round minus the field average strokes gained/lost for the round is the player's Strokes gained/lost for that round. The sum of strokes gained for each round are divided by total rounds played (Line 202-207).

SG: Around the Green (*X8*): The number of Around the Green strokes a player takes from specific locations and distances are measured against a statistical baseline to determine the player's strokes gained or lost on a hole. The sum of the values for all holes played

in a round minus the field average strokes gained/lost for the round is the player's Strokes gained/lost for that round. The sum of strokes gained for each round are divided by total rounds played (Line 220-225).

SG: Putting (*X13*): The number of putts a player takes from a specific distance is measured against a statistical baseline to determine the player's strokes gained or lost on a hole. The sum of the values for all holes played in a round minus the field average strokes gained/lost for the round is the player's strokes gained/lost for that round. The sum of strokes gained for each round is divided by total rounds played (Line 240-244).

**Minor Weaknesses**

①Premise of the Research Gap

***Re:*** To strengthen the justification for focusing on Olympic courses, we explained that Olympic course differ from LPGA tournament course in introduction. Le Golf National, the venue for the 2024 Paris Olympic golf tournament, posed a uniquely rigorous challenge for elite female golfers, characterized by significantly narrower fairways (25-28 yards vs. the LPGA Tour average of 33-36 yards) and rough area maintained at 3.5 inches—substantially higher than the tour standard of 2.5 inches. This course configuration, combined with faster green speeds, prioritized driving accuracy and strategic course management, distinguishing the Olympic competition context from conventional LPGA Tour events.In the realm of golf, capturing quantitative metrics of swing mechanics, shot accuracy, and course strategy has become an indispensable tool for players, coaches, and analysts (Line 58-67).

②Writing and Clarity

***Re:*** We have undertaken a linguistic revision of the entire manuscript, with special attention to the Results section. We have broken down long, complex sentences, clarified awkward phrasing, and ensured a more logical flow. The results are now presented in a clearer, more direct manner, focusing on the key findings from the correlation analysis and the revised regression model (Line 340-359&396-405).

**Responses to Specific Comments：**

Responses to Abstract

***Re:*** We thank the reviewer for this important comment regarding the need for cautious interpretation of model fit statistics. We have comprehensively revised the Abstract to address this concern: We replaced "stepwise regression" with "LASSO regression with LOOCV and bootstrap validation," explicitly communicating the methodological safeguards implemented to address overfitting concerns inherent to small-sample analyses. In the revised abstract, we report "adjusted R² of 0.928 with cross-validated MSE of 0.528," providing both training set performance and cross-validated error estimates for balanced interpretation. We added the qualifying statement "though external validation is warranted," directly addressing the reviewer's concern that high R² values in small samples require cautious interpretation and independent confirmation. Rather than reporting regression coefficients that may be sample-specific, we now highlight bootstrap selection frequencies (≥90%) for seven stable predictors, emphasizing the robustness of variable selection rather than potentially inflated effect sizes.

Responses to Data Availability

***Re:*** In accordance with PLOS ONE's data availability policy, we have now uploaded the all raw data underlying our study as a Supporting Information file (labeled 'Supplementary Material 1') with the resubmitted manuscript (Line 247).

Responses to Introduction

***Re:*** Page 8, Lines 169-173, the positioning you indicate may not be accurate.We have added key discussions on the structural validity and reliability of performance indicators in the introduction. It is critical to emphasize that the scientific application of performance indicators hinges on their construct validity (ability to accurately measure the intended technical or strategic constructs) and reliability (consistency of measurements across contexts), as is standard in sport-specific assessment development (Line 67-71).

Responses to Methods

***Re:*** 1.Sample size limitations, the Women's Golf competition at the 2024 Paris Olympics had only 60 spots available. To ensure the research question was relevant to "elite performance," we focused on the top 10 (including ties) competitors. The scarcity of the group makes it difficult to obtain a larger sample; see Major Weaknesses①Statistical Power and Sample Size for details.

2. we have made substantial revisions accordingly. First, we replaced stepwise multiple linear regression with LASSO regression, which is specifically designed for small-sample, high-dimensional settings through its L1 regularization penalty that shrinks less important coefficients toward zero, thereby mitigating overfitting risks. We employed LOOCV to determine the optimal regularization parameter (λ), maximizing the use of available data while providing honest prediction error estimates. Additionally, bootstrap resampling was performed to assess variable selection stability, with predictors selected in≥90% of iterations considered highly stable—this approach quantifies uncertainty and guards against capitalizing on chance correlations. Regarding PCA, we have removed the dependent variable from the analysis (addressing the reviewer's methodological concern) and now employ PCA solely as an exploratory, descriptive technique to understand the latent structure among the 13 predictor variables. We have also expanded the limitations section to acknowledge that external validation is warranted before definitive conclusions can be drawn.

Responses to Results

***Re:*** The result part of the CV calculation error is corrected, and the relevant data is re-verified to ensure the accuracy of the data. We perform PCA re-correction (Line 313-323&340-359) and linear model construction of lasso (Line 396-405). We sincerely thank the reviewer for this insightful comment. We acknowledge that the original stepwise regression approach was inappropriate given our sample size (n=48) and number of predictors (p=13), potentially leading to overfitting and inflated R² values. In the revised manuscript, we have replaced stepwise regression with LASSO regression, which is specifically designed for situations where the number of predictors approaches the sample size. See Responses to Methods.

Responses to Discussion

***Re:***

1.Page 17, Lines 611-616.

We sincerely thank the reviewer for this insightful suggestion. We have now explicitly incorporated this point in the revised Discussion: “Recent research has demonstrated that biomarker responses can differ significantly between competitive match conditions and standardized testing environments, even within the same athletes [Slimani M, et al.2025;11(1):2447399]. In the unique environment, that is, the data collected in the Olympic Games, it cannot be directly applied to the tour, but it is still of reference significance for the national team's training camp and preparation for the Olympics.” (Lines 414-419).

2.Page 18, Lines 362-364

We fully agree with the reviewer that our previous wording risked overstatement. We have completely rewritten the relevant section to adopt a much more cautious and statistically honest tone. Key changes include: Emphasized the small sample size (n=48) relative to 13 predictors.

Clearly stated that the high adjusted R² (0.928) reflects training-set performance and is likely optimistic. Highlighted the use of LASSO+LOOCV and bootstrap selection frequency as mitigation strategies rather than proof of generalizability. Explicitly described the model as “exploratory” and in need of external validation.

3.Page 19, Lines 388-390

We have substantially expanded and strengthened the limitations paragraph to explicitly acknowledge threats to both internal validity and model stability: A major limitation of this study is the relatively small sample size (48 rounds from 12 players). The limited number of observations relative to the number of predictors poses a direct threat not only to external generalizability but also to internal validity and the stability of multivariate models. Even with LASSO regularization and LOOCV, overfitting and selection instability remain genuine risks in such settings. Consequently, the identified predictors and their estimated effect sizes should be viewed as preliminary until replicated in larger samples (Line 545-552).

4.Page 19, Lines 716-718

We have merged and significantly expanded the kinematic limitation:

The current analysis is restricted to performance outcomes without any insight into how these outcomes were produced. Recent advances in wearable IMU sensors, markerless motion capture, and AI-driven biomechanical analysis now permit detailed, field-based assessment of swing mechanics, ground reaction forces, and muscle activation patterns in elite golfers To explain why certain players achieve superior Driving Distance or Approach proximity, future Olympic or Tour studies should incorporate these technologies. Similarly, integrating electromyography with musculoskeletal modeling of the golf swing that would enable investigation of muscle synergy adaptations and their relationship to performance and injury risk in elite female golfers [Souaifi M, et al.2025;12(8):887; Tajik R, et al.2025;7:1596670] (Line 557-561).

We believe these revisions fully address the reviewer’s concerns and substantially strengthen the revised manuscript. We are deeply grateful for the careful and constructive feedback that has markedly improved the manuscript.
