# Supplementary material for: Correlation of single-round strokes & performance indicators: Paris Olympic elite female golfers
Source: PLoS One. 2026 Jun 5;21(6):e0338350. doi: 10.1371/journal.pone.0338350 (PMC13240931; doi:10.1371/journal.pone.0338350)
Supplement: Attachment — Submitted filename: Response_to_reviewers_auresp_2.docx [file pone.0338350.s004.docx]

**Response Letter**

Manuscript ID: PONE-D-25-49380

Manuscript Title: *Correlation of Single-Round Strokes & Performance Indicators: Paris Olympic Elite Female Golfers*

Dear Academic Editor and Reviewer#1

Thank you for your letter and the reviewer’s constructive feedback on our manuscript. We sincerely appreciate the time and effort dedicated to evaluating our work, and we are grateful for the opportunity to revise and improve the manuscript. All comments have been addressed point-to-point below, with responses highlighted in red font. Moreover, we have indicated the specific locations of the corresponding changes in the revised manuscript. Page and line numbers refer to the clean version of the revised manuscript.

We believe these revisions address all the points raised by the editor and reviewer, significantly improving the quality and clarity of our manuscript. Thank you again for your valuable input. We look forward to your favorable reconsideration of our submission.

Best regards,

The Corresponding Author

**Responses to Reviewer #1**

**Major Weaknesses**

①Table and Figure Labeling

***Re:*** We apologize for the confusion. After deleting the original Table 1 (participant demographic information) to protect privacy, we re-numbered all tables sequentially. The descriptive statistics are now presented as Table 1, and all in-text citations have been corrected accordingly (page 6 line 217; Table 1 caption). We have verified that all table references (Tables 1-4) now match their captions.

②Mathematical Formatting

***Re:*** To avoid ambiguity and readability in SG, we have followed the official definition of the Paris Olympics. We avoid using too many mathematical formulas, as they can make the article harder to read.

③Typos

***Re:*** Thank you to the reviewers for carefully correcting the typos in the manuscript. We have corrected all four instances (lines 90 & 228 & 330 & 346).

**Minor Weaknesses**

①Keyword Consistency

***Re:*** We have replaced “Linear Regression” with “LASSO regression” in the keywords section (line 35). The revised keywords are: “Paris Olympics; Elite Female golfers; Competitive Performance; Principal Component Analysis; LASSO regression”.

②Reference Formatting

***Re:*** We have carefully checked and standardized all in-text citations and reference entries to comply with the target journal’s citation style. Inconsistencies in author names, publication years, journal titles, volume/issue/pages, and DOI formats have been uniformly corrected. Please see the revised reference (pages 14-16).

**Responses to Specific Comments：**

Responses to Abstract

***Re:***

**(1)** We have revised the abstract to include the sample size. The sentence now reads: “Model performance yielded an adjusted R² of 0.928 with cross-validated MSE of 0.528 based on 48 rounds from 12 players, though external validation is warranted.” (page 1 lines 29-31).

**(2)**The keyword “Linear Regression” has been removed and replaced with “LASSO regression” (page 1 lines 35).

Responses to Introduction

***Re:***

**(1)** We have checked citation 17. The original citation addressed general golf course biodiversity, not Olympic course design. We have replaced it with a more appropriate reference (Petrosillo et al., 2019 was removed). Citation 9: Zoffer M. Competitive Golf: How Longer Courses Are Changing Athletes and Their Approach to the Game (page 3 line 100).

**(2)** We have changed “construct predictive models” to “construct a predictive model” to reflect that only one final LASSO regression model is presented (page 3 line 110).

Responses to Materials & Methods

***Re:***

**(1)** Since Table 1 was deleted for privacy protection, we have removed the in-text reference to Table 1 and adjusted subsequent table numbering to maintain logical sequence.

**(2)** To avoid ambiguity and readability in SG, we have followed the official definition of the Paris Olympics.

SG: Off the Tee (*X3*): The number of strokes a player takes from a specific distance off the tee on par 4 and par 5 is measured against a statistical baseline to determine the player's strokes gained or lost off the tee on a hole. The sum of the values for all holes played in a round minus the field average strokes gained/lost for the round is the player's Strokes gained/lost for that round. The sum of strokes gained for each round are divided by total rounds played (page 5 lines 165-170).

SG: Approach the Green (*X6*): The number of Approach the Green strokes a player takes from specific locations and distances are measured against a statistical baseline to determine the player's strokes gained or lost on a hole. The sum of the values for all holes played in a round minus the field average strokes gained/lost for the round is the player's Strokes gained/lost for that round. The sum of strokes gained for each round are divided by total rounds played (page 5 lines 176-181).

SG: Around the Green (*X8*): The number of Around the Green strokes a player takes from specific locations and distances are measured against a statistical baseline to determine the player's strokes gained or lost on a hole. The sum of the values for all holes played

in a round minus the field average strokes gained/lost for the round is the player's Strokes gained/lost for that round. The sum of strokes gained for each round are divided by total rounds played (page 5 lines 184-189).

SG: Putting (*X13*): The number of putts a player takes from a specific distance is measured against a statistical baseline to determine the player's strokes gained or lost on a hole. The sum of the values for all holes played in a round minus the field average strokes gained/lost for the round is the player's strokes gained/lost for that round. The sum of strokes gained for each round is divided by total rounds played (page 6 lines 198-202).

Responses to Results

***Re:***

**(1)** All table numbers in the text have been synchronized with table captions to ensure full consistency (lines 217 & 240 & 251 & 276).

**(2)** “signal round strokes” has been corrected to “single round strokes” (page 7 line 228).

**(3)** The lengthy text-based PCA linear combination equations (Y1-Y5) have been removed from the main text to improve readability. Key component interpretations are now supported by the Feature Vector table (Table 3) as suggested.

**(4)** The dependent variable single-round strokes (Y) was excluded from PCA to avoid circularity and overfitting. The incorrect statement about Y having a high eigenvector has been deleted, and the description of the second principal component has been revised to match the actual PCA results.

Responses to Discussion

***Re:***

**(1)** “signal round strokes” has been corrected to “single round strokes” (page 10 line 330 & page 11 line 346).

**(2)** “its’” corrected to “its” (page 12 line 424).

**(3)** We have consolidated the sentences. The revised: “Future research should employ wearable technology and AI-driven motion analysis to uncover biomechanical drivers of performance, as these tools are increasingly critical for understanding why certain players achieve superior outcomes.” (page 12 lines 409-412).
